# Research progress in brain-targeted nasal drug delivery

**DOI:** 10.3389/fnagi.2023.1341295

**Published:** 2024-01-17

**Authors:** Qingqing Huang, Xin Chen, Sixun Yu, Gu Gong, Haifeng Shu

**Affiliations:** ^1^Department of Anesthesiology, The General Hospital of Western Theater Command, Chengdu, China; ^2^College of Medicine, Southwest Jiaotong University, Chengdu, China; ^3^Department of Neurosurgery, The General Hospital of Western Theater Command, Chengdu, China

**Keywords:** nasal administration, brain targeting, drug delivery, blood–brain barrier, biomaterials, research progress

## Abstract

The unique anatomical and physiological connections between the nasal cavity and brain provide a pathway for bypassing the blood–brain barrier to allow for direct brain-targeted drug delivery through nasal administration. There are several advantages of nasal administration compared with other routes; for example, the first-pass effect that leads to the metabolism of orally administered drugs can be bypassed, and the poor compliance associated with injections can be minimized. Nasal administration can also help maximize brain-targeted drug delivery, allowing for high pharmacological activity at lower drug dosages, thereby minimizing the likelihood of adverse effects and providing a highly promising drug delivery pathway for the treatment of central nervous system diseases. The aim of this review article was to briefly describe the physiological structures of the nasal cavity and brain, the pathways through which drugs can enter the brain through the nose, the factors affecting brain-targeted nasal drug delivery, methods to improve brain-targeted nasal drug delivery systems through the application of related biomaterials, common experimental methods used in intranasal drug delivery research, and the current limitations of such approaches, providing a solid foundation for further in-depth research on intranasal brain-targeted drug delivery systems (see Graphical Abstract).

## Graphical Abstract

According to the factors that affect the absorption of drugs into the brain through the nose, the bioavailability of drugs administered through the nasal brain pathway can be improved by changing the dosage form of the drug, adding mucosal adhesives, applying osmotic enhancers, adding vasoconstrictors, and using a new drug delivery system. 
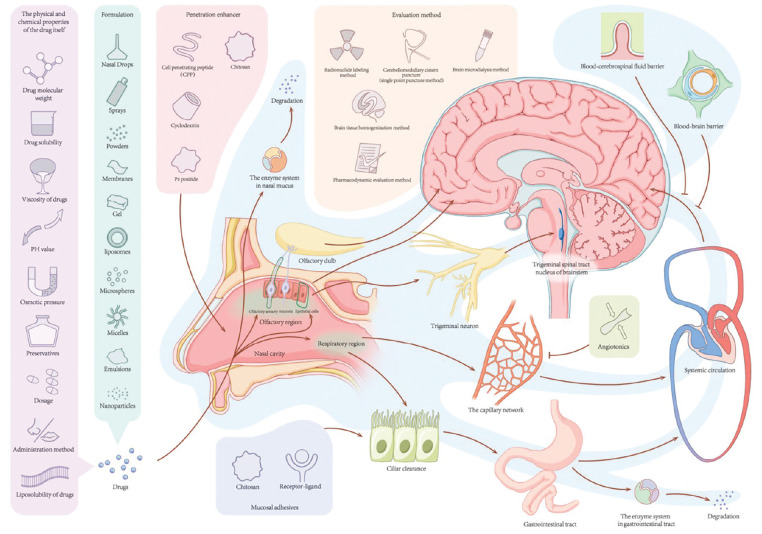

## 1 Introduction

In recent years, the incidence rate of brain diseases such as Alzheimer’s disease, Parkinson’s disease, stroke, and brain tumors has continued to increase, which has been hugely detrimental to human health (Palmer, 2010). These changes have led to increased research activity to develop better therapeutic strategies; however, there are many obstacles affecting the development of new drugs for the treatment of such diseases, one of which is the existence of the blood–brain barrier (BBB) (Palmer, 2010; Xue et al., 2023). The BBB is a complex multicellular structure with extremely low permeability, which limits the movement of molecules between the blood and the neural tissues comprising the central nervous system (CNS) to help maintain homeostasis (Xue et al., 2023). However, the tight junctions of the BBB and the presence of efflux transporters and metabolic enzymes limit the entry of approximately 98% of chemical drugs and nearly 100% of macromolecular drugs (including peptides and genetic drugs) into the brain when administered orally or via injection, making it difficult to ensure such drugs reach the target neural tissues at the concentrations required to induce therapeutic effects; this problem has continued to be a bottleneck that negatively impacts drug delivery in the CNS (Nguyen et al., 2021). Therefore, exploring novel drug delivery systems and pathways to overcome the limitations imposed by the BBB to allow for targeted drug distribution in the CNS has become a top priority for future research.

Nasal administration is a systemic route of administration that leads to rapid drug absorption and high bioavailability, while minimizing the likelihood of adverse effects; these factors, in combination with the ease of administration, have led to increasing research interest in intranasal drug delivery methods (Landis et al., 2012). There is a direct anatomical pathway between the nasal cavity and brain, and studies have shown that some drugs can be administered intranasally, allowing the BBB to be bypassed to facilitate drug entry into the brain, providing a promising approach to brain-targeted drug delivery (Pardeshi and Belgamwar, 2013).

The aim of this review article was to summarize what is currently known about the physiological structure of the nasal cavity and brain, the pathways through which drugs can enter the brain through the nose, factors that affect brain-targeted nasal drug delivery, methods to improve brain-targeted nasal drug delivery through the application of related biomaterials, common experimental methods used in intranasal drug delivery research, as well as current limitations to provide a reference for researchers in the field.

## 2 Physiological structure of the nasal cavity and brain

### 2.1 Physiological structure of the nasal cavity

The human nasal cavity system is a complex structure, with an approximate length of 12–15 cm (Hu et al., 2023). It is composed of two cavities, each of which is approximately 7.5 mL in volume, separated by the nasal septum, with an inner surface covered by mucosa; the mucosa is composed of the epithelium and lamina propria and is approximately 150 cm^2^ in total surface area (Hu et al., 2023). It is generally believed that the average pH of nasal mucus ranges from 5.5 to 6.5 (Hu et al., 2023). The nasal cavity can be divided into three distinct areas based on function and organizational structure, including the vestibular, respiratory, and olfactory areas (Babu et al., 2023). The vestibular area is located in the most anterior part of the nasal cavity and is covered with squamous epithelial cells and contains nasal hair secretory glands that limit drug penetration into the tissue; thus, this area is not typically regarded as a site of drug absorption (Xinchen et al., 2023). The epithelial cells of the respiratory area, which occupies 80%–90% of the inner surface of the nasal cavity, consist of goblet, ciliated, intermediate, and basal cells, and the area contains a large number of mucus-secreting glands and trigeminal nerve branches (Boyuklieva et al., 2023). The olfactory area is located at the top of the nasal cavity, below the cribriform plate (Wu et al., 2023). The epithelial cell layer of the olfactory area is composed of olfactory, supporting, and basal cells. Olfactory cells are bipolar neurons; at one end, there is a dendrite that expands into segments and divides into many cilia that extend into the mucosal layer to allow for communication with the external environment; at the opposite end, there is an axon that can pass through the basal membrane and become wrapped in olfactory sheath cells, ultimately merging into the olfactory nerve, passing through the ethmoid plate and entering the olfactory bulb of the CNS (Awad et al., 2023). In addition, the interstitial fluid surrounding the olfactory nerve bundle is connected to the cerebrospinal fluid (CSF) in the subarachnoid space, and supporting and basal cells can differentiate into new olfactory neurons and other cell types. The lamina propria of the olfactory and respiratory areas contain an abundance of blood and lymphatic vessels that extend to the lymph nodes deep within the neck (Shrewsbury, 2023).

### 2.2 Relevant physiological structure of the CNS

Three protective capsules surround the brain. The outermost layer is the dura mater, with dural sinuses that contain venous vessels, and the innermost layer is the pia mater. The arachnoid membrane exists between the inner and outer layers, with the CSF flowing in the interstices below it (Kadry et al., 2020). CSF is produced by the choroid plexus and flows within the ventricles and subarachnoid space, eventually entering the dural sinuses and mixing with venous blood (Kadry et al., 2020). When the CSF flows through the sieve plates, up to 5% can enter the lamina propria of the olfactory mucosa through the perivascular space near the olfactory filaments before infiltrating into the lymphatic vessels of the nose (Engelhardt and Sorokin, 2009). CSF plays an important role in protecting the brain, maintaining intracranial pressure, and regulating brain metabolism. Although there is an abundant blood supply in brain tissue, a tight BBB is formed by the presence of atresia bands between the endothelial cells of the brain capillaries, an intact basement membrane, and an astrocyte membrane coating the exterior of the vessels. In contrast, the barrier between brain tissue and the CSF, which is composed of soft meninges and glial membranes, is less effective at minimizing the movement of molecules, providing a physiological basis for the pathway of drug delivery from the nose to the CNS via the CSF (Illum, 2004).

## 3 The pathways through which drugs can enter the brain through the nose

At present, the precise pathways through which drugs are transported to the brain following absorption across the nasal mucosa remain unclear; however, four main approaches have been identified to date.

### 3.1 Olfactory nerve pathway

Some studies have shown that most neurophilic viruses (such as rabies, herpetic stomatitis, and equine encephalomyelitis viruses), steroid hormones, metal ions (such as cadmium and nickel), and proteins enter the brain through the olfactory nerve pathway (Crowe et al., 2018). After crossing the olfactory mucosa, these substances are absorbed at the axon terminals of olfactory neurons through pinocytosis, endocytosis, or simple diffusion (Chung et al., 2022). They subsequently flow through the axonal plasma of neurons and are directly transported through the sieve plates to the olfactory bulb, reaching the rhinencephalon (Chung et al., 2022). The olfactory nerve pathway is considered to be the most important pathway for the entry of drugs into the brain from the nose, and it represents the most direct pathway for bypassing the BBB (Ruigrok and de Lange, 2015). However, axonal transport is relatively slow, the rate of which can vary from 0.1∼4 mm/d to 20∼400 mm/d depending on the properties of the drug being administered. Thus, this pathway is characterized by slow absorption, preventing a drug from entering the brain quickly. At the fastest rate, a drug could enter the brain within 1–2 h, however, at the slower rates, some drugs may not enter the brain for up to 24 h, limiting the clinical applications (Singh and Shukla, 2023).

### 3.2 Olfactory mucosal epithelial pathway

The mucosal epithelial pathway (i.e., the nose–brain pathway) is the route through which drugs reach the olfactory mucosal epithelium and enter the CNS directly via cytosolic action or diffusion. Most small-molecule drugs, such as lidocaine, dopamine, 5-fluorouracil, dihydroergotamine, and insulin, are absorbed into the brain via this pathway (Illum, 2003). This pathway is further divided into two components, including a transcellular transport pathway (Illum, 2003) and a paracellular pathway (Wolburg et al., 2008); in the former, drug molecules are transported across mucosal epithelia other than olfactory neuron receptor cells via mechanisms involving carrier transport or cytosolic or passive diffusion into supporting and glandular cells surrounding the olfactory nerve. In the latter pathway, drug molecules enter the intercellular fluid through either the interstitial space of the supporting cells or the peripheral cleft between the supporting cells and the olfactory nerve. If the drug molecules transported to the basement membrane are close to the axons in the lamina propria, they will be transported to the CSF within the cells surrounding the neuron. Simultaneously, drugs in the lamina propria may enter the lymphatic and systemic circulation. If the drug molecules transported to the basement membrane pass through the lamina propria, they will enter the space around the olfactory nerve bundle before being transported into the CSF. This pathway is dependent on the anatomical connections between the olfactory submucosa and subarachnoid space (Ladel et al., 2019). Compared to the rate of absorption via the olfactory nerve pathway, the olfactory mucosal epithelial pathway allows for more rapid drug absorption, facilitating drug entry into brain tissue and the CSF within just a few minutes after nasal administration (Yokel, 2022).

### 3.3 Trigeminal nerve pathway

The ophthalmic and maxillary nerve branches of the trigeminal nerve can extend to epithelial cells in the olfactory and respiratory areas of the nasal cavity, with the opposite end entering the CNS at the pons and terminating at the spinal nucleus of the trigeminal nerve in the brainstem or passing through the ethmoid plate and terminating at the olfactory bulb area (Schaefer et al., 2002). In a study conducted by Thorne et al. (2004), strong radioactivity was observed in the trigeminal nerve, trigeminal branch ganglia, and olfactory bulb following intranasal administration of iodine-125-conjugated insulin-like growth factor 1, with a concentration 10 times higher in the trigeminal nerve than in the olfactory bulb, confirming that, in some cases, the trigeminal nerve may serve as a pathway through which drugs can enter the brain following intranasal administration. However, the transit time along the trigeminal nerve has been reported to be 17–56 h longer than that along the olfactory nerve (Gänger and Schindowski, 2018).

### 3.4 Blood circulation pathway

Low-molecular-weight lipophilic drugs predominantly enter the brain following absorption into the general circulation through the rich capillary network in the lamina propria of the respiratory region. However, after entering the general circulation, drugs must cross the BBB to reach the CNS; thus, this pathway is a limiting factor in the therapeutic application of many drugs (Illum, 2000).

Following nasal administration, a drug will eventually reach the CNS through one or more of the aforementioned pathways, with differences in drug properties, formulations, and routes of administration dictating the dominant pathway of a drug delivery system.

## 4 Factors affecting brain-targeted nasal drug delivery

After a drug is administered via the nose, some of it may enter the CNS directly, whereas some will be absorbed into the general circulation, and some will be removed by the cilia of the nasal mucosa. The amount of drug that can be absorbed through the nasal mucosa is mainly influenced by the physiological characteristics of the nasal cavity, the nature of the drug itself, and factors related to its formulation.

### 4.1 Physiological properties of the nasal cavity

#### 4.1.1 Mechanism of mucociliary clearance

One of the main limitations associated with nasal absorption is the rapid removal of a drug from the nasal cavity by ciliated cells. The cylindrical cilia lining the surface of the nasal mucosa sway at a speed of 5∼6 mm/min in order to move mucus from the nasal cavity to the pharynx and to eliminate substances adhered to the surface of the nasal mucosa. Although this process is a protective mechanism for maintaining optimal respiratory system functionality, it can also shorten the contact time between a drug and the nasal mucosa, directly affecting the efficiency of drug absorption through the nose, as a drug molecule typically remains within the nasal cavity for a period of approximately 20–30 min after administration (Du et al., 2023). To counteract the effects of this clearance mechanism, it is usually possible to extend the drug adhesion time and increase drug absorption by selecting appropriate dosage forms, using bioadhesive materials, or modifying the surface of drug carriers, such as nanoparticles (Badran et al., 2023), liposomes (Kannavou et al., 2023), and microemulsions (Pailla et al., 2022) with specific ligands.

#### 4.1.2 Expression of transport proteins

Efflux mechanisms involving transport proteins expressed in the nasal mucosa, such as multidrug resistance protein 1, breast cancer resistance protein, and P-glycoprotein (P-gp), can limit the absorption of certain drugs. Therefore, inhibiting the synthesis and expression of these proteins can reduce drug clearance (Chen et al., 2018). For example, a previous study demonstrated that P-gp plays an important role in drug absorption and efflux in the nasal cavity, and treatment with PSC-833, a specific P-gp inhibitor, increased the amount of quinidine (a P-gp substrate used as a model drug) reaching the brain via the nasal nerves (Bors et al., 2020).

#### 4.1.3 Role of enzymes

Many enzyme systems catalyze the biological processes occurring within the nasal mucosa; these systems include cytochrome enzyme subunits, epoxidases, polymerases, peptidases, and proteases, all of which can lead to drug degradation in the nasal cavity, producing a “pseudo-first pass effect” that limits the amount of peptide- and protein-based drugs that might otherwise enter the brain (Oliveira et al., 2016). Administration of enzyme inhibitors can reduce the expression or activity of enzymes, thereby increasing drug uptake (Khatri et al., 2023).

#### 4.1.4 Blood vessel absorption

The abundance of capillaries in the nasal cavity can increase the amount of a drug being absorbed into the bloodstream, resulting in high drug concentrations in the general circulation. However, this also reduces the probability of direct drug entry into the brain and increases the likelihood of side effects (Kumar et al., 2016). The use of vasoconstrictors at the administration site can, to some extent, prevent excessive drug diffusion into the bloodstream, thereby increasing brain-targeted drug delivery.

### 4.2 The physical and chemical properties of the drug itself

#### 4.2.1 Drug molecular weight

Under normal circumstances, a higher molecular weight will lower the efficiency of drug absorption (Fortuna et al., 2014). For lipid-soluble drugs, the rate of absorption across the nasal mucosa is nearly 100%; however, these rates are also affected by the molecular weight of a drug, with the absorption efficiency being significantly reduced when the molecular weight exceeds 1 kDa. For macromolecular water-soluble drugs, the concentration of drugs entering the CSF decreases as the relative molecular weight increases. In addition, the absorption efficiency of polar drugs is affected by the molecular weight; for example, when the molecular weight of a polar drug is less than 300 Da, the absorption efficiency is mostly unaffected by the drug’s physical and chemical properties, whereas the absorption efficiency is highly affected by the physical and chemical properties of a drug whose molecular weight exceeds 300 Da (Appu et al., 2016).

#### 4.2.2 Drug solubility

Before a drug can be absorbed through the nasal mucosa, it must first dissolve in nasal secretions. In terms of composition, water accounts for 90% of the components of nasal secretions, with the remainder comprising small amounts of mucin, proteins, inorganic salts, and lipids, among other factors (Hussain et al., 2023). Therefore, drugs with high water solubility will also be soluble in nasal secretions. However, for drugs with low water solubility, carriers such as cyclodextrins (De Gaetano et al., 2022), microemulsions (Pires et al., 2021), and nanoparticles (Abla and Mehanna, 2023) can be selected as drug delivery vectors, allowing the carrier instead of the drug to come into direct contact with the nasal mucosa. The shells of these carriers have good hydrophilicity, which improves their solubility in mucus.

#### 4.2.3 Liposolubility of drugs

The smooth and rapid absorption of drugs across the mucosal layer is dependent on their liposolubility. Usually, drugs with high lipid solubility have good compatibility with the nasal mucosa and are more likely to be absorbed. However, the high fat solubility of a drug can also be detrimental as it can facilitate entry into the general circulation after absorption through the nasal cavity, which is not conducive for drug entry into the brain (Chen et al., 2022; Tanna et al., 2023).

#### 4.2.4 Drug viscosity

In general, drugs with an appropriate viscosity should be more likely to remain in contact with the nasal mucosa for longer periods of time, thereby improving the absorption efficiency (Bseiso et al., 2022). However, a previous study that explored the effect of viscosity on absorption efficiency of metoclopramide hydrochloride drugs reported that although the retention time of a drug in the nasal cavity was prolonged as the viscosity increased, the absorption efficiency actually decreased (Zaki et al., 2007). Most new drug carriers, such as microspheres, gels, and nanoparticles, have been developed with an appropriate drug viscosity in mind, improving both the retention time of drugs in the nasal cavity as well as the absorption efficiency.

### 4.3 Preparation-related factors

#### 4.3.1 pH value

Both the pH of a nasal preparation and the composition of the surface of the nasal cavity affect the dissolution, absorption, and penetration of a drug, with the optimal absorption occurring when a drug is in a non-dissipative state (Yu et al., 2011). The pH of nasal secretions is approximately 5.5–6.5, and preparations with a pH too far outside this range will irritate the nasal cavity (England et al., 1999). Ideally, the pH of the nasal cavity should be maintained at approximately 4.5–6.5 for optimal buffering.

#### 4.3.2 Osmotic pressure

A change in osmotic pressure not only causes the contraction or relaxation of nasal epithelial cells and affects drug absorption, but it may also exacerbate nasal mucosal edema and reduce the beat frequency of ciliated cells in the nasal cavity (Lemoine et al., 2005). Any drugs administered via the nasal cavity should be isotonic with the nasal mucosa (i.e., equivalent to 0.9% sodium chloride solution).

#### 4.3.3 Formulation

Common formulations currently used for the nasal administration of drugs include nasal drops, sprays, powders, gels, microspheres, membranes, emulsions, liposomes, nanoparticles, and micelles (Khan et al., 2017). The physical properties of a drug delivery system can affect the dissolution and retention time of drugs in the nasal cavity. Nasal sprays generally result in significantly higher bioavailability of a drug compared with that of drugs administered via nasal drops, which are quickly cleared by nasal cilia. In contrast, drugs administered via nasal sprays are mainly deposited within the front portion of the nasal cavity, with only a small proportion being slowly cleared into the throat, thereby extending the retention time of drugs in the nasal cavity, facilitating absorption, and improving the bioavailability (Kumar et al., 2017). Powder-based formulations tend to result in stronger and more prolonged contact with the mucosa than solution-based formulations, leading to higher concentration gradients on both sides of the mucosa, thereby increasing drug absorption and brain bioavailability. Many other new dosage forms, such as gel or microsphere preparations, emulsions, liposomes, nanoparticles, and micelles are also conducive to improving the nasal absorption of drugs (Marcello and Chiono, 2023). In summary, an appropriate dosage form should be selected based on the aforementioned specifications on a case-by-case basis depending on the properties of the drug being administered.

#### 4.3.4 Effects of preservatives

Some commonly used preservatives in nasal preparations include benzalkonium chloride, ethylenediaminetetraacetic acid, ethylparaben, and thiomersal. Improper selection of a preservative can affect the absorption of drugs across the nasal mucosa (Li et al., 2018). Lipophilic preservatives such as ethylparaben can reversibly accelerate or reduce the frequency of movement of ciliated cells in the nasal cavity, whereas polar preservatives, such as benzalkonium chloride, tend to reduce the movement of cilia in the nasal cavity.

#### 4.3.5 Dosage

Typically, a higher dosage of a drug will lead to greater absorption and improve the efficacy. However, higher drug concentrations may irritate the nasal mucosa. Moreover, the volume of the nasal cavity is limited, and a dosage that is too high can lead to overflow from the nostrils or entry into the pharynx, causing discomfort. According to a previous study, the appropriate nasal administration volume is 0.05–0.15 mL, and the maximum volume should not exceed 0.20 mL (Mittal et al., 2014).

#### 4.3.6 Administration method

For humans, the method of administration primarily depends on the dosage form (Krishnan et al., 2017). When using nasal drops, for example, the head should be tilted back at an appropriate angle before the medication is administered. Sprays should be directly administered into the nasal cavity, and a plaster must be applied to the inner wall of the nasal cavity. In experimental animals, medications are usually administered directly.

## 5 Methods to improve brain-targeted nasal mucosal drug delivery through the application of related biomaterials

### 5.1 Penetration enhancers

Following intranasal drug administration, the actual amount of drug absorbed through the nasal mucosa is very limited; therefore, a key research goal is to identify ways to increase this absorption. One means of achieving this goal is through the use of penetration enhancers, which increase the likelihood that drug molecules will be subsequently transport into the brain.

#### 5.1.1 Pz-peptidase

Pz-peptidase has been shown to promote drug permeability, and the enzyme can instantly and reversibly induce the opening of tight junctions (Chikuma et al., 1993).

#### 5.1.2 Cell-penetrating peptides

Cell-penetrating peptides are short peptides that can penetrate the cell and/or nuclear membrane to guide any connected peptides, proteins, or other bioactive molecules into cells (Kamei, 2017). Although the precise transmembrane mechanism of cell-penetrating peptides is not fully understood, the effects are likely mediated via signal transduction pathways and endocytosis (Kamei et al., 2021).

#### 5.1.3 Chitosan

The cations present in the amino groups of chitosan can bind to anions in the mucosa, thereby improving the permeability of epithelial cell membranes and promoting the opening of the tight junctions between epithelial cells and enhancing drug absorption (Shim and Yoo, 2020). Chitosan functions as a mucosal adhesive and has a good safety profile, which has led to it being extensively investigated in recent years for its potential therapeutic applications (Hard et al., 2023).

#### 5.1.4 Cyclodextrins

Cyclodextrins are cyclic compounds composed of D-glucose molecules connected by 1,4-glycosidic bonds; they are water-soluble, non-reducing, molecules that take the form of a white crystalline powder (Rassu et al., 2021). Commonly used α-, β-, and γ-cyclodextrins are composed of six, seven, and eight glucose molecules, respectively (Papakyriakopoulou et al., 2021). The unique spatial structure of cyclodextrins allows them to form inclusion complexes with many substances, especially those that are lipophilic. Thus, cyclodextrins can be used as nasal mucosal absorption enhancers, solubilizers, or stabilizers to promote drug absorption, either directly or indirectly (Papakyriakopoulou et al., 2021).

### 5.2 Mucosal adhesives

A significant limitation of the nasal route of administration is the rapid clearance of drug molecules mediated by mucosal fibers within the nasal cavity. Thus, the use of mucosal adhesives can inhibit the clearance functions of mucosal cilia, increasing the retention time of drugs at the mucosa and effectively enhancing both drug absorption and the bioavailability of drugs in the brain.

#### 5.2.1 Chitosan

As described in several studies, various types of chitosan and hydroxypropyl methylcellulose can also be used as mucosal adhesives to facilitate nasal drug administration (Nižić et al., 2020; Kamali et al., 2023).

#### 5.2.2 Receptor–ligand interactions

To increase the nose-to-brain delivery of nanoscale drugs, selecting appropriate ligands for surface modification of the formulation can increase the affinity between the drug delivery system and the mucosa, leading to enhanced mucosal adsorption. The most commonly used targeting ligands are proteins whose receptors are expressed in the olfactory region, namely lactoferrin (Li et al., 2023) or certain glycoproteins (Pernet et al., 2023). Several lectins, such as wheat germ agglutinin (Su et al., 2020) and solanum tuberosum lectin (Chen et al., 2012), have also been used to promote nose-to-brain drug delivery.

### 5.3 New drug delivery systems

The identification of novel drug delivery systems capable of increasing the absorption of drugs across the nasal mucosa and promoting drug transport to the brain have become a research hotspot in the field of targeted drug delivery in the CNS. Some of these systems can achieve their effects without irritating the nasal mucosa, and many have a good safety profile.

#### 5.3.1 Liposomes

Liposomes are novel dosage forms of targeted drug delivery systems. Liposomes are enclosed bilayer membranes of phospholipids (such as lecithin and cholesterol) that contain hydrophilic cores and possess the characteristics and functions of biofilms (Duong et al., 2023). Lipid-soluble compounds can be embedded in phospholipid bilayer membranes, whereas water-soluble compounds can be encapsulated in hydrophilic parts; therefore, liposomes can carry both lipid- and water-soluble drugs (Duong et al., 2023). Owing to their surface charge, liposomes increase the contact time with the mucosa and improve the bioavailability of drugs while also protecting encapsulated biomolecules from degradation by enzymes in the nasal mucosa (Semyachkina-Glushkovskaya et al., 2022). Liposomes cause only slight or no damage to the nasal mucosa, without inducing irritation or ciliary toxicity, and they are suitable for long-term administration (Semyachkina-Glushkovskaya et al., 2022).

#### 5.3.2 *In situ* gel preparations

An *in situ* gel, also known as an *in vivo* gel, is a polymer that can undergo a phase change at the drug delivery site into a liquid or semi-solid form (Tang et al., 2011). Such preparations can effectively extend the retention time of drugs in the nasal cavity and increase drug concentrations in brain tissue (He et al., 2008). Depending on the factor that induces the phase change, *in situ* gels can be classified as temperature-, pH-, and ionic-type gels (He et al., 2008). These gels have a highly hydrophilic three-dimensional network structure, which increases the absorption of drugs across the nasal mucosa by increasing the water permeability. The gels can induce their effects without damaging the mucosal surface because the drugs are transported through the paracellular pathway along with the flow of water (Agrawal et al., 2020). Therefore, in recent decades, researchers have been focusing on optimizing the properties of these gels and improving the understanding of the processes through which they exert their effects.

#### 5.3.3 Microsphere preparations

Microspheres are a new dosage form developed in recent years involving a particle dispersion system formed by drug dispersion and absorption in a polymer matrix (Rassu et al., 2018). Microspheres are a spherical drug delivery system composed of various materials, including but not limited to, albumin, gelatin, polylactides, and starches (Rassu et al., 2018). Microspheres act as strong bio-adhesives and can extend the retention time of drugs in the nasal cavity to 4 h (Pandey et al., 2020). In addition, they can protect drugs from enzymatic metabolism, thereby greatly enhancing their bioavailability (Pandey et al., 2020).

#### 5.3.4 Emulsions

An emulsion is a heterogeneous dispersion system composed of two or more immiscible or partially miscible liquids (Gizurarson, 2012). The particle size of a microemulsion ranges from 10 to 100 nm, they have a transparent appearance, and they contain surfactants and cosurfactants in a thermodynamically and dynamically stable system (Froelich et al., 2021). Microemulsions are capable of carrying large amounts of lipophilic and water-soluble drugs while simultaneously protecting them from degradation, hydrolysis, and oxidation (Froelich et al., 2021).

#### 5.3.5 Nanoparticles

Nanoparticles are drug delivery systems with a particle size of 10–100 nm that are generated using polymer materials as carriers to adsorb or encapsulate drugs within the carrier material (Antunes et al., 2023). Nanoparticles can protect encapsulated drugs from degradation, preventing their unintended removal from the nasal cavity, extending the residence time of drugs in the nasal mucosa, and promoting the direct transport of certain drugs from the nasal cavity to the brain, without increasing drug concentrations within the general circulation; thus, nanoparticles can help facilitate brain-targeted drug delivery (Ferreira et al., 2023). Although the precise mechanism remains unknown, the transportation of nanoparticles to the brain may involve receptor-mediated endocytosis into cerebral capillary endothelial cells (Montegiove et al., 2022).

## 6 Common experimental methods used in intranasal drug delivery research

Currently, there is no suitable *in vitro* method for evaluating brain-targeted drug delivery following nasal administration, and most existing evaluation methods are based on pharmacokinetic or pharmacodynamic techniques.

### 6.1 Cerebellomedullary cistern puncture (single-point puncture method)

The procedure for cerebellomedullary cistern puncture is as follows: after a certain period of drug, administration, the skin on the dorsal side of the head and neck of the mouse is cut open, the foramen magnum is exposed, and a syringe is inserted into the cerebellomedullary cistern to extract the CSF, from which the drug content is quantified (Šakić, 2019). However, due to insufficient CSF supplementation post-extraction, normal intracranial pressure is difficult to maintain, as it is affected by the CSF volume. This method can only be used to determine the CSF concentration of a single mouse at a certain time point after drug administration, and it cannot be used to obtain complete data to characterize the changes in drug concentrations in the CSF over time. It is also difficult to distinguish differences in drug distribution within brain tissue using this method. Because of the need for a large number of animals for experimental purposes, this method has been utilized less frequently than others in brain-targeted drug delivery research (Zhang et al., 2023).

### 6.2 Brain tissue homogenization method

In the brain tissue homogenization method, the whole brain of an animal is collected post-drug administration according to a predefined experimental timeline, the meninges and blood stains are removed, different brain tissues (such as the olfactory bulb and cerebellum) are separated, and the drug content is measured after weighing, homogenization, and sample pretreatment (Onaolapo et al., 2023). This method allows researchers to assess the distribution of drugs in brain tissue at specific time points after drug administration; however, to minimize the impact of individual variability in experimental animals, a large sample size is generally required. Despite this limitation, this method remains one of the most widely used in experimental research (Ramos et al., 2023).

### 6.3 Radionuclide labeling method

The radionuclide labeling method uses isotope labeling to quantify drug content in tissue after administration, making it a suitable technique for studying the brain distribution of peptides or proteins after nasal administration (Elsharkawy et al., 2023). This method allows for rapid detection with high sensitivity, and it does not require tedious drug extraction steps after tissue homogenization, reducing the risk of experimental errors. However, this method cannot distinguish between raw materials, degradation products, and conjugates, making it impossible to determine the true concentration of a drug from the measured total radioactivity (Janssen et al., 2023).

### 6.4 Brain microdialysis method

The microdialysis method is a rapidly evolving *in vitro* or *in vivo* brain chemistry samplingtechnique developed in recent years; it offers good temporal and spatial resolution for determining the concentration of free drugs in the CNS. Compared to traditional research methods, it does not alter the total amount of CSF; thus, it can be performed without affecting the normal physiological function of experimental animals, and it allows for continuous sampling and quantification in a single animal (Zhu et al., 2023). The technique can also be used to determine the concentration of drugs in different brain tissues as well as changes in the concentration of non-binding drugs in the brain’s extracellular fluid over time. This technology has become indispensable in the study of brain-targeting drugs and their active pharmacological effects in the CNS, and it is suitable for *in vivo* biochemical and deep brain region research (Venturini et al., 2023). However, this method has high instrument requirements and costs, and it is not conducive to large-scale experiments.

### 6.5 Pharmacodynamic evaluation method

When a drug concentration is difficult to measure, its known pharmacological effects can be assessed to indirectly infer the degree of drug absorption into the brain through the nasal mucosa.

## 7 Brain-targeted nasal drug delivery for the prevention and treatment of CNS diseases

Brain-targeted nasal drug delivery has broad application prospects in the prevention and treatment of CNS diseases. By administering drugs through the nasal cavity, drugs can directly enter the brain, improve their efficacy, and reduce adverse reactions. Meanwhile, brain-targeted nasal drug delivery can also improve patients’ cognitive function, emotional state, and quality of life. Therefore, brain-targeted nasal drug delivery will become one of the important means for the prevention and treatment of CNS diseases in the future. The application of brain-targeted nasal drug delivery in different CNS diseases is summarized as follows (See Table 1).

**TABLE 1:** The application of brain-targeted nasal drug delivery in CNS diseases.

Types of CNS diseases	Representative diseases	Brain-targeted nasal specific drug delivery
Movement disorders	Parkinson’s disease	Haloperidol (Mohammad et al., 2023), Rotigotine (Saha et al., 2023), Gangliosides (Fuchigami et al., 2023), Rasagiline (ElShagea et al., 2023), etc.
Cognitive dysfunction diseases	Alzheimer’s disease	Insulin (Larijani et al., 2023), Oxytocin (Michaelian et al., 2023), Resveratrol (Fonseca-Santos et al., 2023), etc.
Cerebrovascular diseases	Brain stroke	BDNF (Zhou et al., 2023), Valsartan (Sabry et al., 2023), Salvinorin A (Misilimu et al., 2022), etc.
Mental illness	Depression	Sertraline (Silva et al., 2023), Esketamine (Hope et al., 2023), Quercetin (Elkomy et al., 2023), etc.
Neurological diseases	Epilepsy	Perampanel (Meirinho et al., 2023), Carbamazepine (Bonaccorso et al., 2023), Midazolam (Shaikh et al., 2022), etc.

## 8 Discussion and conclusion

Many scholars have conducted studies on the brain-targeting effects of nasally administered drugs, with aims that have ranged from identifying the mechanism of action to improving drug dosage forms. However, it is still necessary to rationally assess nasal administration systems, as the following are some important limitations that have yet to be overcome: 1) Low targeting efficiency is currently the biggest and most common problem in brain-targeted drug delivery research. The efficiency of such targeted drug delivery systems is usually only several times higher than that of clinically administered drugs or conventional non-targeted drug delivery systems. 2) At present, brain targeting studies mainly focus on ways to improve drug delivery to the brain, with fewer studies focusing on how drugs are distributed after they enter the brain. The structure of brain tissue is complex, and the organ is central to the control of most bodily functions, making it difficult to investigate drug concentrations in various regions in humans. The ability to maximize the concentration of a therapeutic drug at the site of a specific lesion after entry into the brain is of great clinical significance. 3) The use of excipients, receptors, or carriers to enhance drug delivery to the brain can inadvertently affect the metabolism and distribution of endogenous substances, and long-term use of certain medications may cause serious toxic side effects. 4) There are significant anatomical differences between experimental animals and humans, and the results of studies based on animal models may differ significantly from those of studies involving humans. For example, in experimental studies, animals are often anesthetized, which results in low sensitivity to nasal mucosal stimulation and enhanced drug absorption. 5) The effectiveness of brain targeted nasal administration in treating central nervous system diseases is easily influenced by the experimental animal model and the age of the experimental animals. 6) The physiological condition of the nasal cavity has a significant impact on the absorption of drugs. Nevertheless, nasal administration of drugs in order to bypass the BBB and facilitate direct entry of a drug into the CSF or brain tissue is still a safe, convenient, and non-destructive method of targeted drug delivery to the CNS. To optimize the clinical utility of brain-targeted nasal drug administration strategies, it will be necessary to further increase the volume of drugs that can be transported through the nose into the brain, to reduce the significant variability in drug absorption that is caused by physiological changes in the nasal cavity, and to reduce the irritation and long-term toxicity of nasal preparations.

## Author contributions

QH: Writing – original draft. XC: Writing – review and editing. SY: Methodology, Supervision, Writing – review and editing. GG: Writing – review and editing. HS: Funding acquisition, Writing – review and editing.
